# Oxygen-Carrying Polymer Nanoconstructs for Radiodynamic Therapy of Deep Hypoxic Malignant Tumors

**DOI:** 10.3390/biomedicines9030322

**Published:** 2021-03-22

**Authors:** Sandhya Clement, Anna Guller, Saabah B. Mahbub, Ewa M. Goldys

**Affiliations:** 1 ARC Centre of Excellence in Nanoscale Biophotonics, The Graduate School of Biomedical Engineering, University of New South Wales, Sydney 2052, Australia; s.mahbub@unsw.edu.au (S.B.M.); e.goldys@unsw.edu.au (E.M.G.); 2 Institute for Regenerative Medicine, Sechenov University, 119991 Moscow, Russia

**Keywords:** radiodynamic therapy, oxygen-carrying polymer nanoparticles, hypoxia, pancreatic cancer, 3D tumor model in vitro

## Abstract

Radiodynamic therapy (RDT) is an emerging non-invasive anti-cancer treatment based on the generation of the reactive oxygen species (ROS) at the lesion site following the interaction between X-rays and a photosensitizer drug (PS). The broader application of RDT is impeded by the tumor-associated hypoxia that results in low availability of oxygen for the generation of sufficient amounts of ROS. Herein, a novel nanoparticle drug formulation for RDT, which addresses the problem of low oxygen availability, is reported. It consists of poly (lactic-co-glycolic acid) (PLGA) nanoparticles (NPs) co-loaded with a PS drug verteporfin (VP), and the clinically approved oxygen-carrying molecule, perfluorooctylbromide (PFOB). When triggered by X-rays (4 Gy), under both normoxic and hypoxic conditions, PLGA–VP–PFOB nanoconstructs (NCs) induced a significant increase of the ROS production compared with matching PLGA–VP nanoparticles. The RDT with NCs effectively killed ~60% of human pancreatic cancer cells in monolayer cultures, and almost completely suppressed the outgrowth of tumor cells in 2-weeks clonogenic assay. In a 3D engineered model of pancreatic cancer metastasis to the liver, RDT with NCs destroyed ~35% of tumor cells, demonstrating an exceptional efficiency at a tissue level. These results show that PLGA–VP–PFOB is a promising agent for RDT of deep-seated hypoxic tumors.

## 1. Introduction

Photodynamic therapy (PDT) is a clinically recognized minimally-invasive treatment of malignant tumors such as cancers of the skin, head and neck, and urinary tract surfaces, as well as intraperitoneal carcinomatosis and sarcomatosis [1]. PDT relies on the destruction of the cancer cells and stimulation of other anti-tumor mechanisms by highly cytotoxic reactive oxygen species (ROS) generated via the interaction of a particular class of drugs, the photosensitizers (PS), with ultraviolet, visible, or near-infrared light. The PS absorb the energy of the light and transfer it to the neighboring oxygen molecules or other molecular species, which then can react with available oxygen. This results in the formation of free radicals, including highly toxic singlet oxygen (^1^O_2_) that play a central role in the therapeutic mechanism of PDT [2,3].

There are three major limitations for a broader application of conventional PDT in oncology. The first one arises from the fact that the locally advanced solid malignant tumors are usually severely hypoxic [4], with O_2_ tensions <2.5 mmHg [5]. This prevents efficient ROS generation in PDT, as most of PSs require notably higher (pO_2_ ≥ 8–16 mmHg) levels of molecular oxygen in the tissue [6]. Moreover, PDT draws down the oxygen existing in the tissue and thus it aggravates local hypoxia [7]. Additional deficiency of the conventional PDT is that due to limited depth penetration of light its effect is mostly superficial, and it can destroy tumor tissue only to a depth from few mm up to 1.5 cm from the treated surface [1,3,8]. The third challenging aspect of PDT is the current limited control over the properties of PSs (e.g., bioavailability and target specificity) and their biological effects [2].

In the current work, we address these shortfalls of traditional PDT in the context of the potential treatment of a deeply located hypoxic malignant tumors with limited conventional treatment options or resistance to them. One of the most prominent examples of such cancers is pancreatic ductal adenocarcinoma (PDAC), an aggressive gastrointestinal malignant neoplasia with a median patient survival of about 6–8 months after diagnosis [9]. PDAC is usually diagnosed very late when it tends to be unresectable, while alternative therapies (chemo- and chemoradiotherapy) seem to have limited efficacy [10,11]. This cancer is exceptionally oxygen-deficient comparing to the healthy tissues and most of the other solid cancers [4,12]. Both the primary PDAC tumors developing in the pancreas and the metastatic pancreatic carcinomas which tend to disseminate to the liver [13], are located several centimeters away from the body surface, i.e., at the depth unreachable by the PDT. As a first step to overcome these challenges, we developed a novel nanoscale drug formulation allowing the simultaneous delivery of an oxygen-carrying molecule with a PS triggerable by a clinically relevant dose of X-rays for the efficient generation of ROS. Since clinical X-rays, in contrast to light, can penetrate throughout the human body, the deeply located pathological structures can be targeted. Such approach is termed X-ray mediated PDT (X-PDT), or radiodynamic therapy (RDT) [14,15,16].

Here, a clinically used drug verteporfin (VP) [17,18] was employed as an X-ray triggerable PS with efficient ROS generation [19]. The perfluorooctylbromide (PFOB, C_8_F_17_Br) was chosen as an oxygen-carrying perfluorocarbon molecule [20,21]. To overcome the hydrophobicity of these compounds and achieve their co-delivery, we embedded both, VP and PFOB, into biocompatible and biodegradable [22] poly(lactic-co-glycolic acid) (PLGA) nanoparticles (NPs). The resulting formulation is referred here as PLGA–VP–PFOB nanoconstructs (NCs). In the present study, the uptake and intracellular localization of the NCs, ^1^O_2_ generation, and in vitro RDT effects in monolayer cell cultures of human PDAC cell line PANC-1 are shown under normoxic and simulated hypoxic condition. Next, the treatment effects are validated via the tumor cells clonogenic assay and live/dead assay in three-dimensional (3D) engineered tumor models of the early hepatic metastases of pancreatic cancer.

## 2. Materials and Methods

### 2.1. Materials

Resomer^®^ RG 504 H; Poly(D,L-lactide-co-glycolide) (PLGA; #719900), poly(vinyl alcohol) (PVA; #363138), dichloromethane (DCM; #650463), Verteporfin (VP; #SML0534), perfluorooctylbromide (PFOB, or 1-Bromoheptadecafluorooctane; #343862), acetone (#179124), dimethyl sulfoxide (DMSO; # D2650), human PDAC cell line (PANC-1), Dulbecco’s Modified Eagle’s Medium-high glucose without L-Glutamine (#D5671), Gentian violet (#G2039) were purchased from Sigma-Aldrich, North Ryde, NSW, Australia. Singlet Oxygen Sensor Green probe (SOSG; #S-36002), NucBlue™ Live ReadyProbes™ Reagent (#R37605), LIVE/DEAD™ Viability/Cytotoxicity Kit for mammalian cells (#L3224), MitoTracker™ Green FM (#M7514), LysoTracker™ Green DND-26 (#L7526), DPBS, with calcium, magnesium (#14040182), DPBS, with no calcium, no magnesium (#14190250), TrypLE™ Express Enzyme, with no phenol red (#12604021), L-Glutamine, 200 mM (#25030081) and Fetal Bovine Serum, qualified, US origin (#26140079) were purchased from Life Technologies, Mulgrave, VIC, Australia. CellTiter 96^®^ AQueous One Solution Assay was purchased from Promega, Sydney, NSW, Australia.

### 2.2. Synthesis of PLGA–VP and PLGA–VP–PFOB NCs

The PLGA–VP–PFOB NCs were synthesized using a single emulsion solvent evaporation method with a minor modification as described elsewhere [23]. Briefly, a 3 mM VP stock solution was prepared in chloroform (~2.2 mg/mL). The 200 µL of VP stock solution (2.2 mg/mL) and 100 µL of PFOB stock (1.93 g/mL) were added to 30 mg of PLGA solution prepared in chloroform (2 mL). Then this mixture was added to 12.5 mL of 5% PVA solution (in water) and sonicated using a probe sonicator for 1.5 min (3 × 30 sec). After each 30 sec of sonication, the mixture was placed on ice to cool down for 1 min. Then the resulting NC suspension was stirred at 1000 rpm for 4 h at room temperature to get rid of the chloroform. Then the equilibration of the NCs suspension was performed in a dialysis against either the DI water or PBS for the further experiments. In order to prepare the PLGA–VP NCs, the same steps were repeated except the addition of PFOB.

### 2.3. PLGA–VP–PFOB NCs Characterization

The size and the zeta potential of the NCs were measured using Zetasizer Nanoseries (Malvern Instruments, Malvern, Worcestershire, UK). All the measurements were performed 3 times at 25 °C. The fluorescence (Excitation: 405 nm, Emission: 650–750 nm) from VP in the NCs was measured using FluoroMax plus C (JY Horiba, Kyoto, Japan) by using a quartz cuvette at room temperature. To confirm and quantify the amount of VP, the absorption spectra of the NCs was analyzed by Cary 5000 UV-VIS-NIR absorption spectrophotometer (Varian Inc., Palo Alto, California, USA) using paired 1-cm pathlength cuvettes.

### 2.4. Cell Culture

The PANC-1 cells were cultured in a complete cell culture medium (CCM) prepared with DMEM (without L-glutathione) mixed with 10% FBS, 1% antibiotic-antimycotic and 1% L-glutamine. Cells were grown at 37 °C with a 5% CO_2_. When the cells reached 80% confluency, they were transferred either to the dishes or to the 96-well plates according to the experimental requirements.

### 2.5. Viability Assay (Dark Toxicity Study)

In order to check the viability of the cells in the presence of PLGA–VP–PFOB NCs without light and radiation exposure, cells were seeded in 96-well plates and incubated with or without NCs with different concentrations of VP (for different concentration of VP, we diluted the NC) for 4 hours. The cells treated with NCs were washed with the CCM and incubated for the next 24 hours. Then the cell viability assay (CellTiter 96^®^ AQueous One Solution, an MTS assay) was added according to the vendor’s protocol and the absorbance at 490 nm was measured using a SpectraMax i3x Multi-Mode Microplate Reader (Molecular Devices, San Jose, CA, US).

### 2.6. Irradiation Experiments

All the X-ray irradiation was performed using X-RAD 320, a 320 kVp Orthovoltage Energy X-ray system (PRECISION, North Branford, CT, USA). X-RAD 320 is a self-contained X-ray system designed to deliver a precise radiation dosage to the specimens in a biological or small animal research laboratory. The X-ray tube produces a highly homogenous beam with a maximum power output of 320 kV for fast and accurate dosage specifically for radiation therapy. The samples (either cells or 3D models) were placed inside the sample chamber for the exposure to a specific X-ray dose (4 Gy). For all the RDT experiment in cells, we chose 4 µM of VP (both in PLGA–VP and PLGA–VP–PFOB) concentration in 1 mL CCM for incubation.

### 2.7. Cellular Uptake and Localization of Nanoconstructs

For the analysis of the cellular internalization of NCs, we applied confocal laser scanning fluorescence imaging and digital image analysis.

First, we investigated the minimum time of incubation that NCs are required to be taken up by the cells. PANC-1 cells were seeded into 35-mm Petri dishes and allowed to grow to reach 60% confluency. Next, the cells were added with a fresh portion of CCM containing NCs and incubated for 1, 2, 3 or 4 hours. The control cells were kept in CCM without NCs. Then, the cells were washed three times, added with fresh CCM without NCs and incubated overnight to correspond to the further treatment conditions with X-rays. For the detection of the cellular uptake of NCs, the NucBlue™ Live ReadyProbes™ Reagent was added to stain the nuclei of the cells according to the vendor’s protocol. After that, the cells were imaged using a confocal laser scanning microscope (Olympus FV3000, Shinjuku, Tokyo, Japan) with 405 nm excitation. The emission from the cell nuclear region (430–480 nm) and the fluorescence emitted from the VP (650–700 nm) was detected, and the colocalization of the NucBlue and VP signals was measured using ImageJ 1.52n software (NIH, Bethesda, Maryland, USA).

To define the NCs intracellular localization, the cells were seeded and prepared in the same way as described above, and the incubation with NC was performed for 4 hours. After the incubation, the cells were thoroughly washed with PBS, added with fresh CCM, and incubated overnight. Then the cells were alternatively stained with Lysotracker and MitoTracker probes and Hoechst 33342 for tracking the organelles such as mitochondria, lysosomes, and nuclei. The cells were imaged at 490/513 nm, 504/511 nm and 405/460 nm excitation/emission wavelength ranges for tracking of the mitochondria, lysosomes, and nuclei, respectively. The respective fluorescence signals’ colocalization was quantified using ImageJ software to examine the cellular uptake and intracellular positioning of NCs.

### 2.8. Effects of X-PDT In Vitro

The following assays were used to determine the RDT treatment effects in PANC-1 cells.

#### 2.8.1. Live/Dead Cell Assay

The efficacy of the cytotoxic treatment was examined by a ready-to-use, live/dead cell imaging kit R37609 (Invitrogen, Waltham, MA, USA). This probe contains two different reagents, NucBlue^®^ to stain the nuclei of the live cells (blue staining) and NucGreen^®^ to stain the nuclei of the dead cells (green staining). The cells were seeded in a 35-mm Petri dishes with the initial density of 1 × 10^5^ cells/mL. After the treatments with NCs and X-rays, two drops of both the reagents were added to the dishes with 1 mL of CCM and the cells were imaged after 15 min. The 425 nm excitation/460 nm emission and 488 nm excitation/525 nm emission were used for the detection of the live and dead cells, respectively.

#### 2.8.2. Clonogenic Assays

Clonogenic assay was applied to analyze the long-term proliferation of PANC-1 cells after various treatments (cells only, cells + NCs, cells + X-ray, cells + RDT). Each treatment condition was triplicated. After the treatments, cells (500 cells/35-mm Petri dish) were incubated for 2 weeks with changing the media every other day. After 14th day, the cells were fixed by adding 4% formaldehyde for 15 min at room temperature. Next, the cells were washed with PBS (with calcium and magnesium) and added with 5 drops of Gentian violet in each dish at room temperature. After 30 min, the cells were carefully washed to remove all the excess stains from Gentian violet. Then, the dishes were allowed to dry for another 1 h and the number of colonies (more than 50 cells) were counted per condition with the help of a microscope. The survival fraction was calculated using the formulae [24]:

(1) $$Surviaval\;fraction=\frac{Number\;of\;colonies\;formed\;after\;treatment}{Number\;of\;cells\;seeded\times Control\;plating\;efficiency}$$

Where the control plating efficiency is the percentage of seeded cells that survived to form colonies under control conditions and can be calculated using the formula:

(2) $$Control\;plating\;efficiency=\frac{Number\;of\;colonies\;formed}{Number\;of\;cells\;seeded}\times 100$$

#### 2.8.3. Toxicity Study in Hypoxic Condition

The hypoxia in PANC-1 cells was induced using CoCl_2_ × 6∙H_2_O following the previously published protocol [25]. Briefly, cells were seeded (1 × 10^5^ cells/mL) in a 35-mm Petri dishes. After 24 h, the CCM in the wells was added with 1 mL of 100 µM of CoCl_2_ × 6H_2_O and cells were incubated for the next 24 hours. Next, the cells underwent RDT treatment.

#### 2.8.4. Singlet Oxygen Detection, Quantification, and Analysis

The singlet oxygen detection was carried out by using the SOSG probe. The stock of SOSG (500 µM) was made by adding 660 µL of methanol into the SOSG vial. The cells were seeded (1 × 10^5^ cells/mL) on 35 mm Petri dishes, added with the NPs/NCs (1 mL), incubated for 4 hours, washed and then added with 8 µM of SOSG (in 1 mL CCM) to each dish and incubated for another 1 h. Then CCM was discarded, the cells washed twice with PBS, added with fresh CCM, and exposed to X-ray radiation (4 Gy). After the irradiation, cells were imaged (at 488 nm excitation/525 nm emission) using confocal microscopy. The fluorescence of the SOSG was quantified using ImageJ software.

#### 2.8.5. Experiments on 3D Cell Cultures

To prove the efficiency of the RDT with PLGA–VP–PFOB NCs at a tissue level and in the biologically relevant, organ-specific environment prior further testing in animals, we assessed the cytotoxic impact on the PANC-1 cells using a 3D cell culture model representing of an early-stage metastatic PDAC. This model was created using the principles of tissue engineering. Macroscale (~5–10 mm^3^) constructs were formed by combining PANC-1 cells with solid scaffolds and individually cultured in vitro. The scaffolds were prepared by decellularization of chicken livers obtained from a local meat shop. As a result of this procedure, an acellular liver-specific extracellular matrix (LS-ECM) was obtained. The details on the preparation and characterization of the 3D engineered model of metastatic PDAC are given in the Appendix A, Section A.6.

#### 2.8.6. Statistical Analysis

Statistical analysis was carried out in MATLAB ((R2020a, https://au.mathworks.com/; accessed on 18.03.2021)). The descriptive statistics is presented as the Mean ± Standard Deviation (SD) of the mean and the 95% confidential interval for mean (CI_95%_) from at least three experiments, if not specified overwise. The quantitative data distribution was examined One-sample Kolmogorov-Smirnov test for the further selection of the methods for the statistical hypothesis evaluation. Then, normally distributed data were analyzed by parametric methods (Student’s T-test and ANOVA) and the data with the distribution different from normal was examined by non-parametric methods. For the non-parametric analysis, the Mann-Whitney U (i.e., the two-sided Wilcoxon’s rank-sum) test was applied to identify the statistical significance. The statistical significance of the differences was accepted at *p* ≤ 0.05. The achieved statistical significance in labelled as following: * represents *p* ≤ 0.05, ** represents *p* ≤ 0.01 and *** represents *p* ≤ 0.001.

## 3. Results

### 3.1. Characterization of PLGA–VP–PFOB NCs

**Figure 1:**
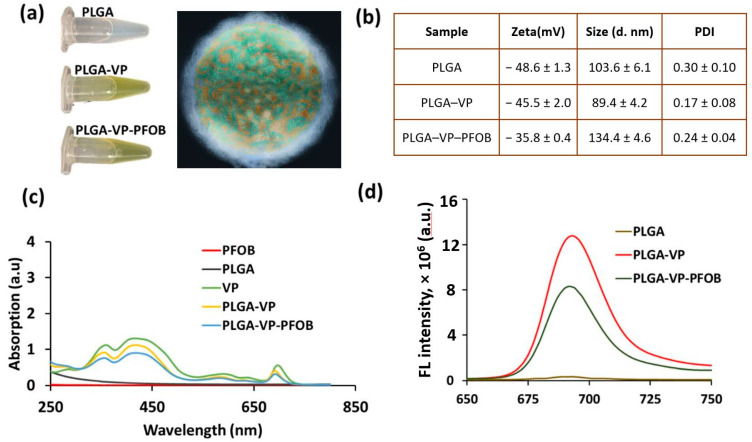
Nanoconstructs (NCs) characterization (**a**) photograph of nanoparticles (NPs) and NCs suspensions in deionized (DI) water and a schematic representation of poly (lactic-co-glycolic acid)—verteporfin-perfluorooctylbromide (PLGA–VP–PFOB) NCs (white “fur”—poly (lactic-co-glycolic acid) (PLGA); green—verteporfin (VP); brown—perfluorooctylbromide (PFOB)); (**b**) zeta-potential, hydrodynamic diameter and polydispersity index (PDI) of the prepared NPs and NCs in DI water; (**c**) absorption spectra of NCs and their components; (**d**) Fluorescence spectra (FL intensity) of PLGA, PLGA–VP and PLGA–VP–PFOB showing the emission from VP with a peak around 700 nm under 425 nm excitation; both absorption and fluorescence spectra are scaled for better visibility).

**Figure A1:**
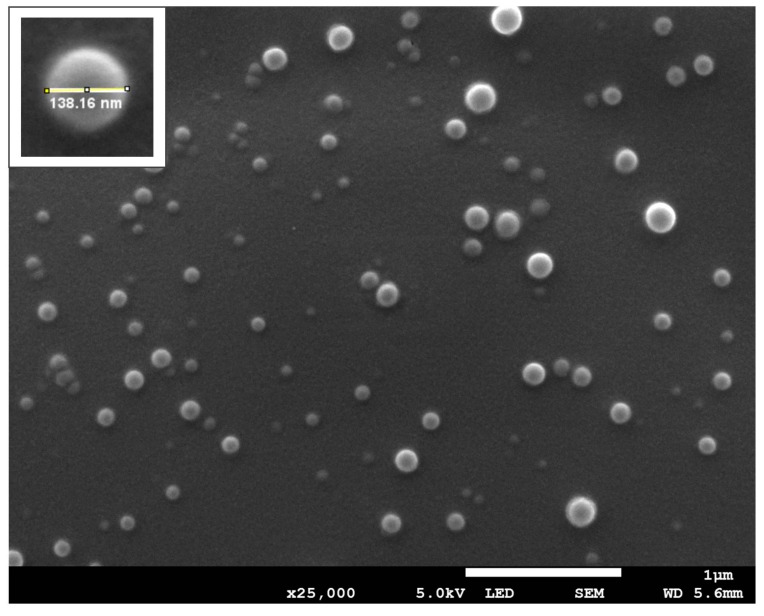
The SEM image showing the shape of the poly (lactic-co-glycolic acid) (PLGA) nanoparticles (NPs). The NPs seem to be spherical in shape. Inset: a zoomed-in view of a single PLGA–VP–PFOB nanoconstruct.

The NCs contained both VP and PFOB molecules co-embedded in the PLGA matrix and all studied particles were stable in aqueous environment (Figure 1a). As it can be seen from Figure 1b, the NPs and NCs had significant negative zeta potentials, and the good polydispersity indexes (indicating monodispersity). The hydrodynamic diameters of the particles were varying between ~90 nm (PLGA–VP) and ~140 nm (PLGA–VP–PFOB). The SEM image (Appendix A, Figure A1) of the PLGA NPs shows that they are mostly spherical. The presence of VP and PFOB molecules inside the PLGA nanoparticles was confirmed using absorption spectra (Figure 1c). Additionally, Figure 1d presents the fluorescence spectra (425 nm excitation/700 nm emission) of VP that was used to monitor the NCs localization in cells and tissues during confocal imaging.

### 3.2. Cellular Uptake and Localization of Nanoconstructs

**Figure 2:**
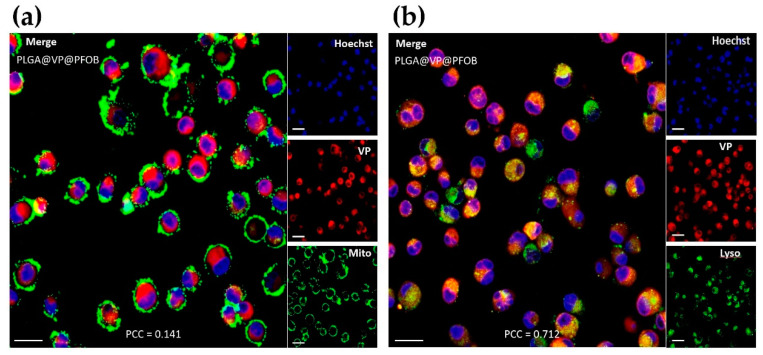
Colocalization of poly (lactic-co-glycolic acid)-verteporfin-perfluorooctylbromide (PLGA–VP–PFOB) nanoconstructs with (**a**) mitochondria and (**b**) lysosomes of PANC-1 cells after 4 h of incubation. Right side panels in (**a**,**b**) show the fluorescence signal of Hoechst (blue, nuclear DNA), verteporfin (VP) (red), and organelles’ trackers (Mitotracker in (**a**) and Lysotracker in (**b**), green). Left side panels are the merged images of all the three stainings depicted separately in the right side panels. PCC is the Pearson’s correlation coefficient indicating the overlap of verteporfin with mitochondria/lysosome. Scale bars are 20 µm.

**Figure A2:**
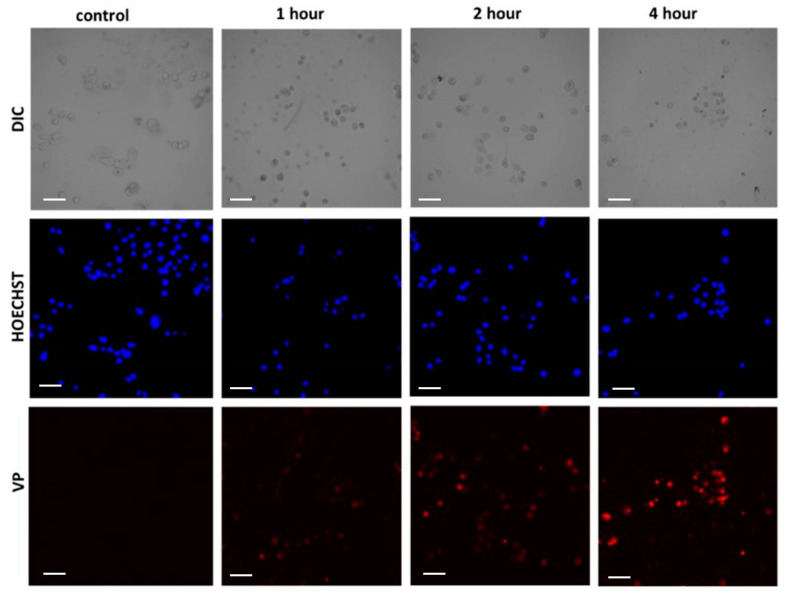
The confocal microscopy image showing the cellular uptake of poly (lactic-co-glycolic acid)- verteporfin-perfluorooctylbromide (PLGA–VP–PFOB) nanoconstructs by PANC-1 cells after 1 h, 2 h and 4 h of incubation. The highest uptake of PLGA–VP–PFOB is observed after 4 h incubation. Untreated cells serve as control. cells. Blue: Hoechst; Red: Verteporfin (VP).

**Figure A3:**
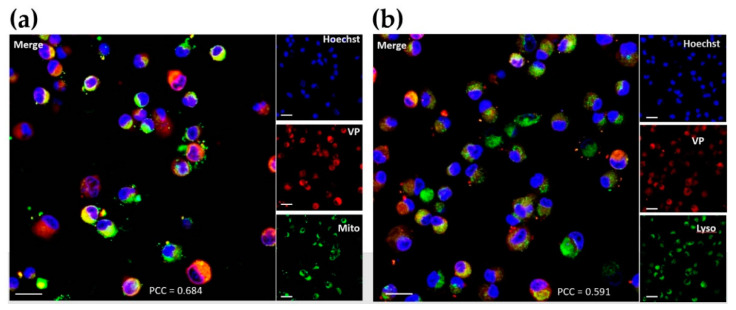
Colocalization of poly (lactic-co-glycolic acid)-verteporfin (PLGA–VP) with (**a**) mitochondria (**b**) lysosomes of PANC-1 cells after 4 h of incubation. Right side panels show the fluorescence signal of Hoechst (blue), verteporfin (red) and mito/lyso tracker (green). Left side images are the merged figures of all the three given in the right panel. PCC is the Pearson correlation coefficient indicating the colocalization of the fluorescent signals of verteporfin (VP) with mitochondria/lysosome trackers.

In a series of preliminary experiments, we found that the 4 h incubation is optimal for most of the NCs to enter the cells and to ensure the maximum availability of the NCs for the therapeutical applications (Appendix A, Figure A2), respectively. Then, the colocalization of PLGA–VP–PFOB NCs with different cell organelles was studied after 4 h incubation. The Figure 2 shows the localization of PLGA–VP–PFOB in mitochondria and lysosomes detected by fluorescence microscopy. There was a larger overlap of fluorescence of VP (red) with lysosomes (Figure 2b) than with mitochondria (Figure 2a) probes. The quantitative analysis of colocalization of VP fluorescence with the Lysotracker and MitoTracker signals confirmed this visual observation. Pearson’s correlation coefficients (PCC) for the lysosomal colocalization were 0.712 vs. 0.141 for the mitochondrial one. In contrast, NPs without PFOB (PLGA–VP) were mainly found in mitochondria rather than the lysosome (Appendix A, Figure A3).

### 3.3. RDT in PDAC Cells under Normoxic Conditions

**Figure 3:**
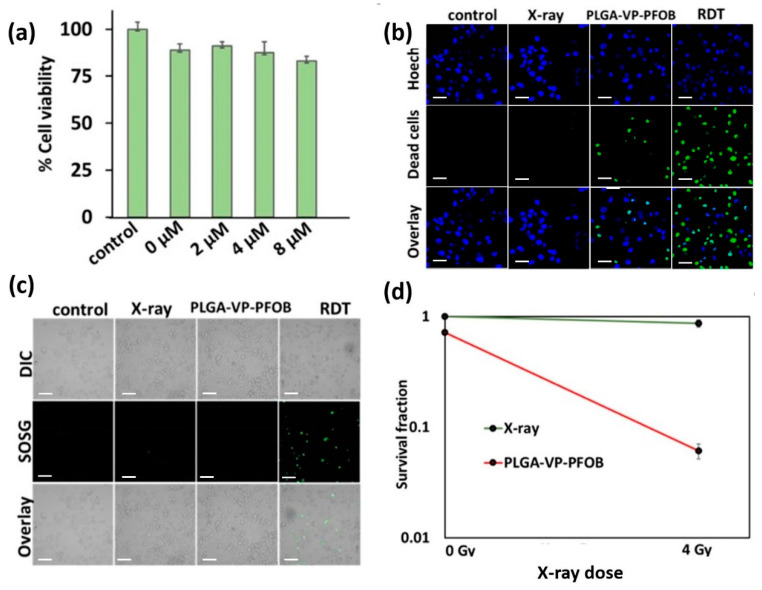
Cell viability analysis under simulated normoxic conditions in monolayer cultures of PANC-1 cells. (**a**) Evaluation of dark toxicity of verteporfin (VP); (**b**) Confocal microscopy imaging of live/dead (blue/green) staining of the cells exposed to the different experimental treatments; (**c**) singlet oxygen generation detection using SOSG (green) assay (**d**) cell proliferation assessment, measured using clonogenic assay after 14 days of post treatment. The red line indicates the change in survival fraction for cells incubated with poly (lactic-co-glycolic acid)-verteporfin-perfluorooctylbromide (PLGA–VP–PFOB) nanoconstructs followed by 0 Gy and 4 Gy of X-ray radiation. The green line indicates the change in survival fraction for the cells treated with 0 Gy and 4 Gy X-ray only. (**b,c**) Scale bars 20 µm.

We found that the dark toxicity of PLGA–VP–PFOB was low, compared to the untreated control (without added NCs). The corresponding cell population loss varied from ~7% to ~17% (Figure 3a) depending on the concentration of VP. Based on the dark toxicity results, we chose, for further studies the NPs and NCs with 4 µM of VP, leading to dark toxicity of 10% under our experimental conditions.

**Figure A4:**
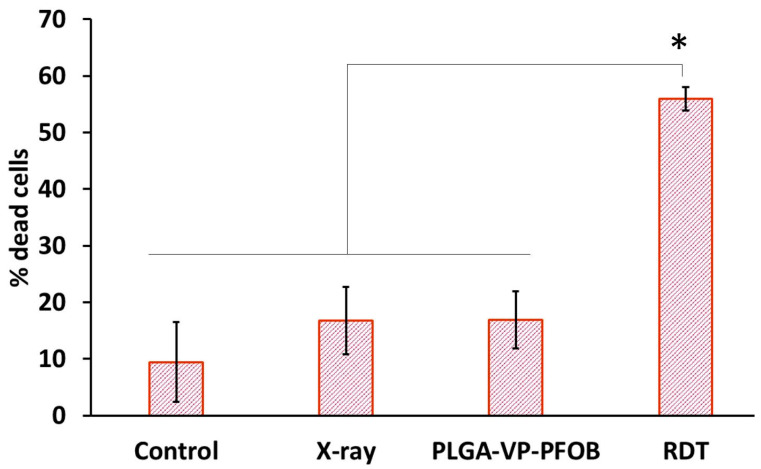
Quantification of dead cells using the results of live/dead cellular assay for different treatment groups (under normoxic conditions). * *p* < 0.05.

According to the results of the fluorescence live/dead assay, there were no statistically significant differences in the fraction of the dead cells between the control (9.47 ± 7.02%), X-ray (16.80 ± 5.97%), and NCs-treated (16.89 ± 5.05%) groups, while the PLGA–VP–PFOB NCs-aided RDT resulted in significant increase of the number of dead cells up to 55.95 ± 2.08%, in comparison to the in vitro cultures exposed individually to equal dose of X-rays and NCs (Figure 3b and Appendix A, Figure A4).

**Figure 4:**
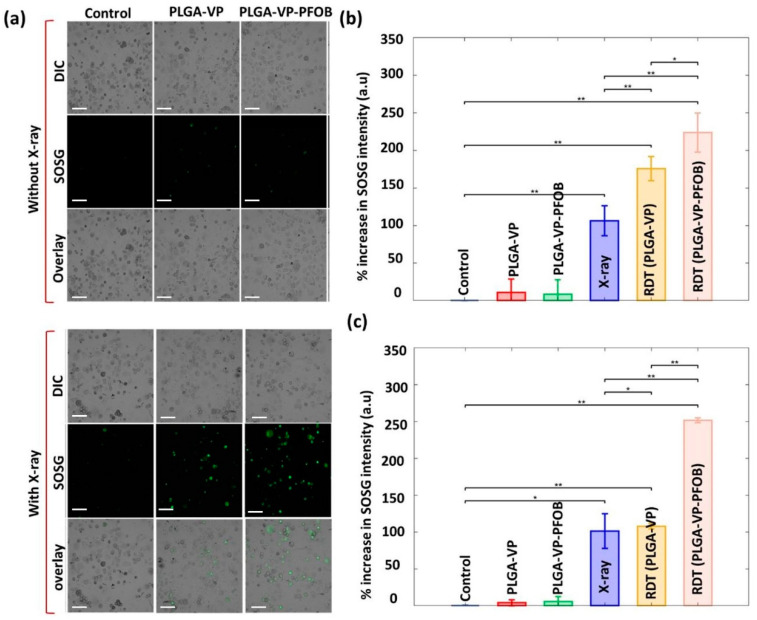
Experiments under modeled tumor hypoxia conditions: (**a**) Singlet oxygen generation from PANC-1 cells with different treatments under modeled hypoxic condition. Scale bars are 20 µm. Quantification of singlet oxygen under (**b**) normal condition and (**c**) hypoxic condition. Here * presents *p* ≤ 0.05, ** represents *p* ≤ 0.01.

Analysis of the singlet oxygen generation (Figure 3c and Figure 4b) revealed statistically significant increase of SOSG fluorescence intensity in X-ray- (107.43 ± 2.84 a.u., CI_95%_ (102.90, 111.95)) and RDT-treaded samples (243.58 ± 60.01 a.u., CI_95%_ (148.09, 339.06)), in comparison with the untreated control (0.58 ± 0.68 a.u., CI_95%_ (0.00, 1.65)) and NC-exposed cell cultures (33.70 ± 44.87 a.u., CI_95%_ (0.00, 105.10)).

Correlation analysis revealed strong positive and statistically significant correlation between the level of ROS generation by the studied treatment modalities and the fraction of dead cells in the treated cultures (the Spearman’s correlation coefficient *Rs* = 0.727, *p* = 0.001, *n* = 16).

**Figure A5:**
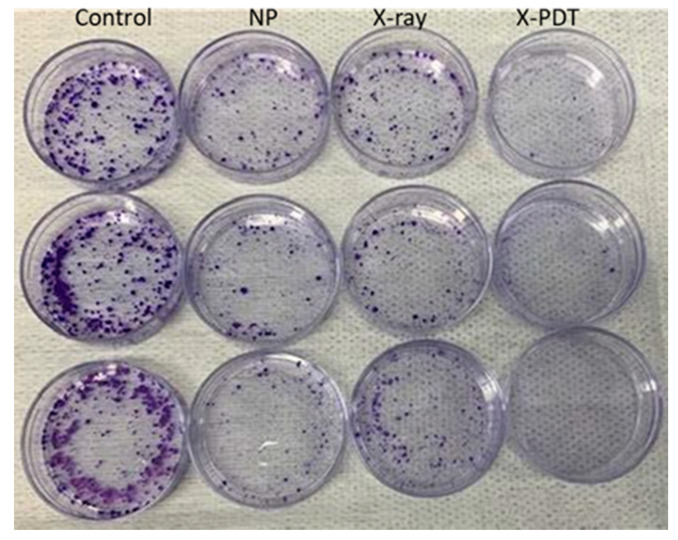
Clonogenic assay results for various treatment groups visualized by Gentian violet staining. Three replicates are shown for each group.

To explore the long-term effect of RDT on PANC-1 cells, we applied a clonogenic assay [26]. This revealed over an order of magnitude difference in the number of surviving colonies of cells which were treated with RDT, compared to the treatment controls and the untreated cells (Figure 3d). Appendix A, Figure A5 shows examples of the stained colonies in the RDT-treated and control groups.

### 3.4. Experimental Treatment under Modeled Tumor Hypoxia

Figure 4a demonstrates the effect of studied experimental treatments performed under hypoxic conditions with clearly visible ROS generation induced by X-rays in PLGA–VP and PLGA–VP–PFOB treatment groups. Further quantification of SOSG fluorescence intensity, the probe of ROS, measured under normoxic and hypoxic conditions is presented in Figure 4b,c, respectively. In normoxia, the ROS generation in the cells treated with PLGA–VP–PFOB was similar to those in PLGA–VP group (*p* ≤ 0.05) after the 4 Gy X-ray irradiation. In contrast, under hypoxic conditions, the amount of singlet oxygen generated from PLGA–VP was very limited due to lack of oxygen, and the SOSG fluorescence signal from the cells treated with this PLGA–VP was comparable with the same signal in cells treated with X-rays only (Figure 4c). At the same time, there was a significant increase in the SOSG fluorescence in cells treated with PLGA–VP–PFOB-aided RDT compared to the RDT with PLGA–VP and other controls (*p* ≤ 0.01) under hypoxia.

### 3.5. RDT in 3D Model

**Figure A6:**
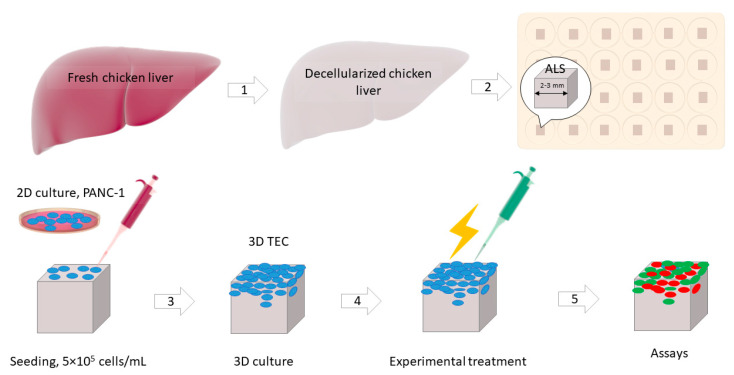
The design of the experiment on three-dimensional (3D) cell cultures of PANC-1 cells. Lobules of fresh chicken liver underwent mild decellularization (1) in agitating baths with a detergent. The decellularized liver the was cut (2) into small fragments (acellular liver scaffolds, ALSs) by a biopsy puncher, sterilized, individually placed in 24-well plates, and preconditioned by the overnight incubation with CCM. Then the ALSs were recellularized by seeding them with PANC-1 cells. The resulting 3D tissue engineering constructs (TECs) were cultured in vitro (3) for 1–30 days after which they underwent experimental treatments (4) and were employed (5) in various assays for characterization or the TECs and function of the cells (SEM, histology, and viability tests).

The 3D tissue engineered constructs (TECs) were created (Appendix A, Figure A6) and employed as experimental testbeds in NCs/RDT study. The protocols used for the preparation and characterization of 3D TECs are presented in Appendix A, (Section A.6.1, Section A.6.2, Section A.6.3, Section A.6.4, Section A.6.5, Section A.6.6 and Section A.7) and shown in Figure A6.

**Figure 5:**
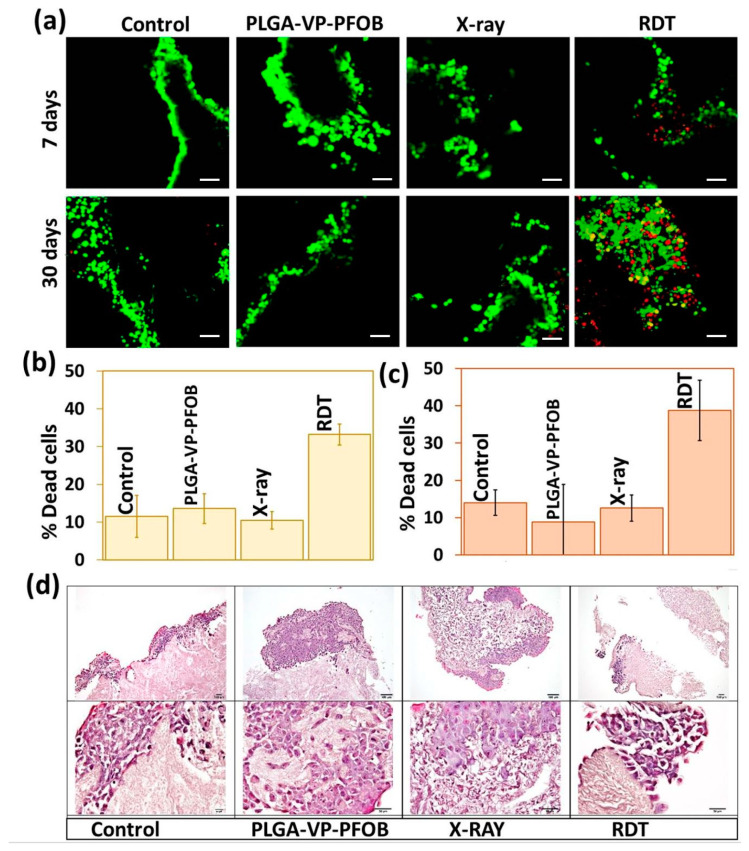
Cytotoxicity study in three-dimensional (3D) tissue engineering construct (TEC) model: (**a**) confocal image showing the live/dead cell staining of the 7-day and 30-day-old TECs that underwent different treatments. Scale bars are 20 µm; (**b**) quantification of % dead cell from the confocal image for 7-day-old TECs and (**c**) 30-day-old TECs; (**d**) Hematoxylin and eosin staining of 7-day-old TECs that underwent different treatment. The upper row demonstrates the overview of the TECs structure at low magnification (scale bars are 100 µm) and the low row of images shows the fragments of the same samples at high magnification (scale bars are 50 µm).

**Figure A8:**
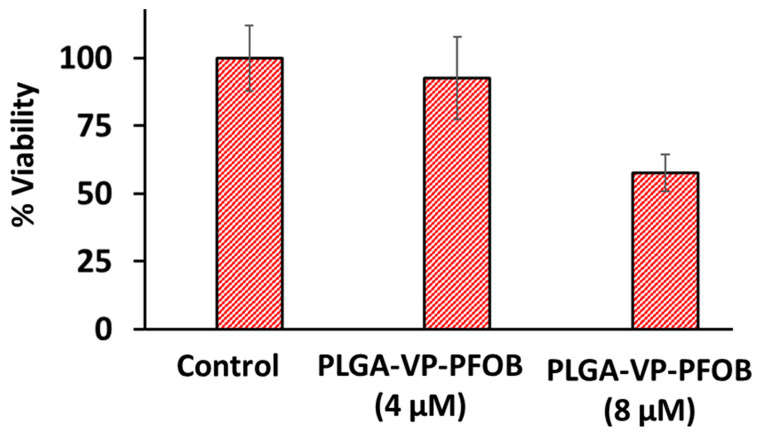
The results of dark toxicity assessment of PLGA–VP–PFOB NCs with different concentrations of VP performed in 3D PANC-1 model using MTS assay.

The dark toxicity of PLGA–VP–PFOB in 3D TECs has been tested for different concentrations of VP (Appendix A, Figure A8). The PLGA–VP–PFOB with the 4 µM concentration of VP was found to be considerably less toxic to the reconstructed PDAC tissue compared with higher VP concentrations. The treatment efficacy was monitored in 7- and 30-day-old TECs using confocal microscopy (Figure 5a). No statistically significant difference in the percentage of cells killed by RDT was found between the 7- and 30-day-old TECs by further image analysis using ImageJ (Figure 5b,c), respectively). The number of dead cells in RDT-treated 3D TECs was around 35%, which is lower, compared to 60% in the case of monolayer PANC-1 cell culture. Figure 5d shows the representative results of histological examination of 7-day-old TEC which underwent various treatments. We found that in the untreated TECs, the cells formed large (400–1000 µm) clusters with solid tumor structure near the surfaces of the LS-ECM scaffolds. In other parts of the TECs, cancer cells formed single- or multirow cellular linings. The external layer of cells covering the large cell clusters and the multirow linings contained flattened and elongated cells. The invasive behavior of cancer cells was noticeable but appeared moderate. In particular, cells permeated in depth of the scaffolds via the pores and voids of the substrate and formed single- or multirow linings of the cavities.

The structure of 7-day-old TECs changed following the experimental treatments. The treatment with PLGA–VP–PFOB without X-ray triggering did not reduce the tumor volume or structure. The TECs treated with X-rays only (4 Gy) demonstrated a notable reduction of the surface tumor masses, and remodeling (loosening) of the ECM. The TECs treated with PLGA–VP–PFOB-aided RDT show the most obvious reduction of the tumor volume (up to 10 times vs. control) and the absence of cancer cells in the depth of the TECs in combination with increased density of the ECM. These findings represent first evidence for the potential therapeutic efficiency of RDT against a tissue-level malignant neoplasia in our model of metastatic pancreatic cancer foci in the liver microenvironment.

## 4. Discussion

In this study, a novel nanoscale RDT agent, the PLGA–VP–PFOB NCs, was explored in order to address the major limitations of conventional PDT such as shallow penetration depth of the light triggering the PS, the insufficiency of oxygen at the lesion site for the efficient ROS generation, and the challenges linked to the delivery of the PS to the tumor cells. The composition of the proposed NCs together with clinical X-rays were chosen to enable accelerated clinical translation.

As a therapeutic compound of NCs, we applied a well-recognized PS drug, VP. Approved by FDA in a liposomal form (known as Visudyne) this drug is indicated for the suppression of angiogenesis in age-related macular degeneration [17,18]. In conventional PDT, VP initiates ROS generation after triggering by the red visible light [1]. In addition to the cytotoxic action of ROS on blood vessels, PDT directly affects cancer cells by destabilization of cell membranes, enhancement of autophagy and apoptosis [27]. The efficiency of VP-aided PDT was demonstrated in various experimental models of mammary carcinoma [28,29], pancreatic [30] and colorectal [23,31,32] cancers, glioblastoma [33] and some other malignancies (see references in [34]). Clinical application of VP for PDT was reported for the eye, pancreatic and skin cancer [1,35]. Clinical trials of VP-aided PDT are currently in place for breast, prostate, pancreatic and brain cancers [36]. Recently, in addition to its role as a PS, new important biological properties of VP which are independent of light activation were discovered. In particular, VP inhibits tumor growth by specific binding to the pro-oncogenic YAP-TEAD protein complex [34,37] that is also involved in upregulation of fibrotic and angiogenic reactions [38] contributing to the tumor advancement and treatment resistance of PDAC [13,30,39]. Next, VP demonstrates a light- and YAP-independent “proteotoxic” mechanism of a specific suppression of proliferation of cancer cells by down-regulation of clearance of high-molecular weight proteins from the cytoplasm [40].

The choice of VP was especially strongly motivated by our own recent results indicating a novel mechanism of light-independent triggering of PS activity of VP by clinically relevant dose of X-rays [19,23,31,32]. Shifting from the visible light to X-rays as initiators of ROS generation (such as PDT to RDT), allows to overcome the tissue penetration depth limit of conventional PDT [14,15,16]. A particular version of this approach based on nanoscale scintillators conjugated with PSs and able to generate cytotoxic ROS under X-ray exposure were demonstrated both in vitro and in vivo [41,42,43]. The direct activation of VP by secondary electrons generated in the tissue following X-ray irradiation allows its application as an X-ray stimulated PS. Preliminary evidence for this has been obtained in our previous work [23,31]. Another possible mechanism is the triggering of VP by the Cherenkov light generated in the tissue [44,45,46]. As both PDT and radiation can kill tumor cells via different mechanisms, the combination of these factors in RDT has the potential to offer synergistic effects and lowers the therapeutic doses of ionizing radiation.

The hypoxia of the tumor tissue is among key factors which lead to a poor survival rate in patients with pancreatic cancer [39]. PDT is less efficient in hypoxic tumors as adequate amount of oxygen cannot be provided to the PS to generate cytotoxic ROS sufficient to kill clinically significant numbers of cancer cells [4]. This dependence of PDT/RDT on the availability of molecular oxygen at the tumor site can be overcome by re-oxygenation strategies. This can be achieved, for example by using oxygen generating agents which produce oxygen by decomposition of the endogenous H_2_O_2_ or the specific compound itself, e.g., MnO_2_ or catalase-based nanoparticles [7]. However, the application of artificial oxygen generating materials remains technically challenging [7]. An alternative option is oxygen carrying molecules such as hemoglobin or perfluorocarbons that solubilize high amounts of oxygen and release the oxygen molecules entrapped with them in a hypoxic environment. Perfluorocarbons are among the most clinically advanced, reliable and popular chemically inert oxygen-carrying molecules suitable for PDT [6,7,21,47,48] because of the stability of their oxygen supply in various environments and significant extension of ^1^O_2_ lifetime [6,47], in comparison to aqueous solutions. In the current work, we employed a perfluorocarbon molecule, perfluorooctylbromide (PFOB, C8F17Br), which has good oxygen-carrying properties, low viscosity and high diffusivity [49]. Its role is to enrich the environment with oxygen and facilitate ROS generation in hypoxic conditions in the tumor [20,21]. This is exceptionally important for the applications in such severely hypoxic tumors as PDAC [4,12]. Recently PFOB was successfully applied for the liposomal co-delivery with a chemotherapy drug in a lung cancer model [50] and in photothermal therapy [51] and PDT [52] in breast cancer cellular xenografts.

It is known that both VP and PFOB may induce some undesirable health effects if acting outside the tumor site [6,29]. This stimulated the development of nanoscale vehicles to ensure improved pharmacokinetics and safety during the delivery of these agents to the disease foci. PLGA nanoparticles (NPs) are widely used in PDT [23,53] due to their fast biodegradability, non-toxicity and almost universal suitability for the delivery of drugs [54] of various chemical nature (including the majority of PS that are also poorly water-soluble) [53,55,56]. Importantly for the anti-cancer use, these NPs can passively accumulate at the tumor sites via the enhanced permeability and retention effect (see [57] for review). An additional advantage of PLGA particles is facile clinical translation following the approval of these biomaterials as a safe drug delivery agent by regulatory bodies such as FDA and European Medicine Agency [22]. Several applications of PLGA NPs as delivery agents for VP [23,29,53] and perfluorocarbons [58,59,60] have been demonstrated. The feasibility of RDT aided by VP-loaded NPs, including PLGA [23] was recently proved by our studies [19,23,31,32,42].

In the current work, we co-embedded VP and PFOB in PLGA NPs and demonstrated the feasibility and efficacy of the PLGA–VP–PFOB NCs as RDT agents in 2D cell- and 3D tissue-level in vitro models of human PDAC. First, we confirmed that the NCs satisfy the essential requirement to the nanoformulated drugs, namely the stability of the dispersions NCs in aqueous environments, which is related to their strongly electronegative surfaces and good PDI. The size of the NCs was within 100–140 nm range (Appendix A, Figure A1) that is considered optimal for the delivery to non-phagocytic cells [61].

We revealed the predominant accumulation of PLGA–VP–PFOB in lysosomes following the 4 h incubation with PANC-1 cells cultured in monolayers (Figure 2). In contrast, PLGA–VP without PFOB after the same exposure time were found almost equally distributed between lysosomes and mitochondria (Appendix A, Figure A3), which is similar with our previous observation of the subcellular localization of these NPs with and without targeting folic acid moieties in colorectal cancer cells [23]. The mechanism of the 4 h stability of the VP fluorescence signal in lysosomes of the cells exposed to the NCs is another feature requiring further studies, as the majority of PLGA nanoparticles are prone to rapidly (in 10 min) escape the endolysomal compartment [62]. The accumulation of the nanoparticles in specific organelles depends on many factors that may affect interactions between the nanomaterial and cell membranes. Therefore, the size and surface properties of the particles play the most important role in this process [63]. According to our data, the PLGA–VP and PLGA–VP–PFOB nanoparticles most notably differed in size and slightly in surface charge and PDI (see Figure 1b). This allows to attribute the predominant lysosomal accumulation of PLGA–VP–PFOB NCs in lysosomes vs. the mitochondrial accumulation of PLGA–VP, at least partially, to the increased size of the NCs, in comparison with the PLGA–VP. It is known that after the uptake by cells, the PLGA nanoparticles are not definitely confined to a single subcellular compartment and depends on the type of epithelial cells [64]. Then, the NCs/PLGA–VP localization data shown in the current study depicts the accumulation of the particles in the specific organelles in PANC-1 cells after 4 h incubation. Importantly, the lysosome accumulation of the NPs loaded with a PS drug allowed enhanced PDT compared to the non-targeted PSs [65]. This property is beneficial for RDT applications of the NCs investigated in the current study.

For the RDT, we applied X-rays in a clinically low dose of 4 Gy [66]. The efficient ROS generation via the interaction of PLGA–VP–PFOB with this ionizing radiation was confirmed by using of SOSG probe specifically sensitive to singlet oxygen one of highly toxic ROS generated during PDT [67]. This ROS generation (Figure 3c) may be attributed to the direct activation of VP by X-rays or due to secondary electron activation of the PS [23,31].

The achieved level of ROS generation was sufficient for killing of ~60% of PANC-1 cells cultured in monolayers and ~35% of the same cells in our innovative tissue engineering 3D model of PDAC metastasis to the liver within short time (15 min) after RDT with PLGA–VP–PFOB NCs. This indicates high efficiency of the proposed therapeutic modality, considering the apoptosis-resistant nature [68] of PANC-1 cells and reduced drug responsiveness of the cancer cells in the scaffold-based 3D cultures [69,70]. The cytotoxic effect of RDT aided with NCs was significantly higher than the dark toxicity (~10–20%) induced by the VP present in the particles.

Finally, we obtained a strong evidence for the specific PLGA–VP–PFOB efficiency as an RDT agent under simulated hypoxia of PDAC cells in monolayer cultures (Figure 4) and in intrinsically hypoxic reconstructed 3D PDAC models (Figure 5). In the experiments on PANC-1 cells monolayers, the oxygen-carrying NCs generated almost twice more amounts of ROS, in comparison with PLGA–VP, while under normoxic conditions the difference in ROS production following X-ray triggering between these nanoscale particles was around 20–30% only. This indicates that PFOB released oxygen in the low oxygen tension environment with supporting enhanced ROS accumulation in the vicinity of the X-ray triggered VP. Our experiments with 3D reconstructed metastatic PDAC tumors demonstrated the efficiency of RDT with the oxygen-carrying NCs at the tissue level in a macroscale (~10 mm^3^) structure. Due to the size of these tumor constructs and absence of vascularization and blood supply, their deep parts represent a naturally hypoxic environment (as the diffusion limit for oxygen in solid cellular aggregates ranges from 100 µm to 200 µm [71]). At the same time, it is known that the tumor cells in 3D culture systems are usually more drug- and radio-resistant to the treatment that the same cells cultured in monolayers [72,73,74]. Following RDT with NCs, approximately 35% of PDAC cells growing in the liver ECM were killed, which, as we think, is a very good and promising result considering the lack of the available treatment options for the locally advanced and metastatic PDAC [11].

The current study is the first step towards the development of the novel therapeutical interventions for the deeply located hypoxic malignant tumors that require the diversification of the treatment modalities. We envisage that the future exploration and development of the sophisticated targeted agents may help to further enhance the efficacy of oxygen-carrying polymer nanoconstructs for RDT.

## 5. Conclusions

In this study, we have demonstrated that the PLGA-PFOB-VP NCs synthesized by the proposed methodology are efficient RDT agents under both, normoxic and hypoxic, conditions. The evidence of the therapeutic efficiency of the NC-assisted RDT was obtained in monolayer cultures of human PDAC cells and in macroscale three-dimensional tissue engineering constructs mimicking the early staged of PDAC metastasis to the liver. The mechanism of the observed cytostatic and cytoreduction effects is attributed to the ROS generation induced in the vicinity of the NCs by a low dose (4 Gy) X-ray irradiation. Our data shows statistically significant (76% vs. PLGA–VP NPs under normoxic and 140% under hypoxic conditions, (*p* ≤ 0.05 for 76% for and *p* ≤ 0.01 for 140%) increase of ROS generation following the supplementation of the PLGA–VP NPs with PFOB molecules under RDT treatment. This indicates the potential of therapeutic application of these NCs in treatment of the clinically challenging deeply located and hypoxic tumors. Additionally, RDT show high cytoreductive efficiency in the tissue engineered hepatic metastases of PDAC, providing an ethical and reliable roadmap for the successful transition to the experiments on animals and later to clinical trials.

## Figures and Tables

**Figure A7:**
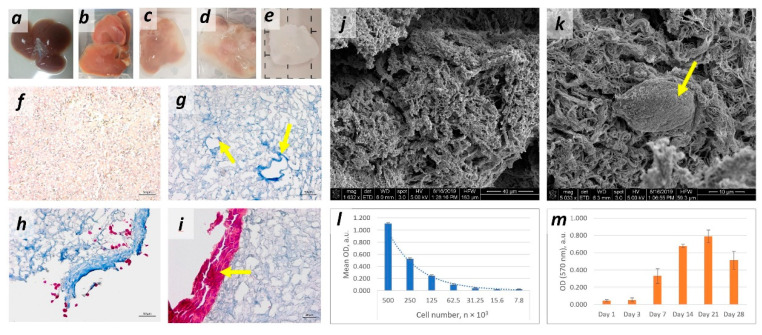
Preparation of acellular liver scaffolds (ALSs) and three-dimensional (3D) tissue engineering constructs (TECs). (**a**–**e**) Changes of the macroscale presentation of chicken liver undergoing decellularization (DCL): (**a**) fresh chicken liver; (**b**) DCL, 4 h, note the overall reduction of the color intensity and a rim of translucent tissue near the surface of the organ; (**c**) DCL, 8 h, note uneven discoloration and formation of the large translucent areas of the tissue; (**d**) DCL, 7 days, extended translucent parts; (**e**) DCL, 10 days, completely decellularized organ, dash lines indicate 1 cm sections; (**f**–**i**) histological examination of the: (**f**) native chicken liver stained by Van Gieson; (**g**) decellularized liver stained by Masson’s trichrome method (MTCh), note absence of cellular staining and blue staining of ECM collagen; arrows show stromal elements (former blood vessels) with denser collagen; (**h**) recellularization of ALS with PANC-1 cells, day 1, note individual cells stained purple by MTCh near the ALS surface; (**i**) recellularization of ALS with PANC-1 cells, 7 days in vitro, note formation of multilayered/solid cellular linings on the ALS surface (arrow), MTCh staining; scale bars in (**f–h**) are 50 µm and in 20 µm (**i**); (**j**–**k**) SEM imaging: (**j**) a surface of ALS, note multiscale textures; (**k**) an individual PANC-1 cell (arrow) attached to the ALS surface in the area with coarse collagen bundles on day 1 in vitro; (**l**–**m**) evaluation of the cell growth dynamics in 3D TECs by MTT assay (*n* = 8 for each examined group): (**l**) calibration of the optical density (OD) vs. PANC-1 cell numbers performed in 2D culture for the relative estimation of cellular density in 3D TECs at different stages of growth; (**m**) MTT assay performed in 3D TECs.

## Data Availability

The data presented in this study are available within the article.

## Appendix A

### Appendix A.2. Cellular Uptake of PLGA–VP–PFOB in PANC-1 Cells

Figure A2 represents the time-based uptake of the poly (lactic-co-glycolic acid)-verteporfin-perfluorooctylbromide (PLGA–VP–PFOB) nanoconstructs by PANC-1 cells.

### Appendix A.3. Colocalization of PLGA–VP with Organelles in PANC-1 Cells

Figure A3 shows the internalization of PLGA–VP by mitochondria and lysosomes. The analysis shows that slightly more nanoparticles ended up in the mitochondria (PCC = 0.684) than in lysosomes (0.591).

### Appendix A.4. Live/Dead Cell Viability Quantification in Monolayer Culture of PANC-1 Cells

Figure A4 shows the quantification of live/dead cell assay results of RDT in cells by digital analysis of the confocal images using ImageJ.

### Appendix A.5. Clonogenic Assay

Figure A5 shows the photograph of Gentian violet staining of the PANC-1 cells colonies for clonogenic assay.

### Appendix A.6. Experiments on Three-Dimensional Cell Cultures

#### Appendix A.6.1. The Experimental Design

The three-dimensional (3D) cultures of PANC-1 cells were established via tissue engineering methodology and employed as models of the early stage of metastatic colonization of the liver by pancreatic cancer cells for the analysis of the effects of the experimental treatment on PANC-1 cells grown in the organ-specific microenvironment. The experimental design is illustrated in Figure A6. The details of the preparation of 3D tissue engineering constructs (TECs) are given below.

#### Appendix A.6.2. Preparation of Acellular Liver Scaffolds (ALS)

Acellular liver scaffolds (ALSs) were prepared from chicken livers obtained from a local poultry supplier. The liver lobules were decellularized as described by us elsewhere with minor modifications [75,76]. Briefly, chicken liver lobules were washed with PBS and placed in 50-mL Falcon tubes, containing 35 mL of sodium dodecyl sulphate (0.1% *v/v* in water). The tubes were then secured on an orbital shaker, agitated at a speed of 90–150 rpm, washing solution was replaced every 3 h for the first 12 h and then every 6 h–12 h until complete decellularization (1–1.5 weeks) (Appendix A, Figure A7). Next, the residual detergent solution was removed from the scaffolds by washing in a 1% (*v/v*) solution of Antibiotic-Antimycotic (Sigma-Aldrich, # A5955) in PBS on sterile water with daily changing of wash solution during 3 days. The resulting ALSs were stored in the fresh portion of the same solution at 4 °C until further use. ALSs preserved the original architecture and composition of the liver extracellular matrix (ECM) (Appendix A, Figure A7).

#### Appendix A.6.3. Recellularization of ALS

ALSs were cut into fragments using a 4 mm biopsy puncher and sterilized with 0.1 (*v/v*) peracetic acid in 4% (*v/v*) ethanol for 2 h at room temperature. Then the sterilization solution was removed, and the ALSs were washed with sterile PBS, placed in 24-well plates, and left under UV for 30 min, then the scaffolds were flipped upside down by sterile tweezers and the UV sterilization repeated for the next 30 min. After sterilization, 1 mL of complete culture media (CCM) was added to each well containing ALS and incubated overnight at 37 °C. Then the media was removed, and ALSs were seeded with PANC-1 cells at a density of 1 × 10^5^ cells/per scaffold/20 uL of CCM. The seeded scaffolds were placed in the tissue culture incubator for the next 2 h to allow attachment of the cells and then added with fresh CCM (1 mL per well). After overnight incubation, the obtained 3D PDAC/ALS tissue engineering constructs (TECs) were transferred into the new 24-well plates in order to avoid the outgrowth of the non-scaffold bound cells on the plastic bottoms of the wells. The average efficiency of cell seeding on the ALSs measured on day 1 in vitro (DIV 1) was 17 ± 2%. For the further 7–30 days, the 3D TECs were grown at 37 °C under a humidified atmosphere of 5% CO_2_/95% air, the CCM changed every 2 days. On the DIV 1 TECs were samples for scanning electron microscopy (SEM), and on DIV 7 they were collected for histological examination. On DIV 7 and DIV 30, the TECs were assayed for in cellular viability after application of the experimental treatments.

#### Appendix A.6.4. Scanning Electron Microscopy (SEM)

The TECs were fixed in 2.5% buffered glutaraldehyde, dehydrated in 70–100% alcohols and underwent critical point drying in Emitech K850 Critical Point Dryer (Emitech Ltd., Ashford, Surrey, UK). Afterwards, the samples were mounted on stabs with conductive carbon/graphite paint (ProSciTech, Kirwan, QLD, Australia) and coated with platinum using an Emitech K550 gold sputter coater (Emitech Ltd., UK). Electron microscope images were taken using a JEOL JSM- 6480 LA under accelerating voltage 5 kV, work distance 20 mm and size point 30 by the secondary electron imaging mode.

#### Appendix A.6.5. Histology

Formalin-fixed tissue samples were processed through alcohols of increasing concentrations and embedded in paraffin. Microtome slices of 5–6 μm in thickness were stained with hematoxylin and eosin (H&E) and Masson’s trichrome method (for collagen). Stained slides were mounted with Depex mounting medium (manufacturer) and covered with glass cover slips. The histological preparations were examined by an upright light microscope BX53 microscope (Olympus, Shinjuku, Tokyo, Japan) equipped with Plan Apochromat 4 × / NA 0.16, 20 × /NA 0.75, 40 × / NA 0.95, and oil-immersion 100 × / NA 1.4 objectives (Olympus). Images were recorded using a digital DP80 camera (1360 × 1024 pixels, Olympus).

#### Appendix A.6.6. Cell Viability Assays in 3D TECs

*MTT assay.* For the analysis of cell growth dynamics in 3D TEC, a modified colorimetric MTT assay was applied. Briefly, this assay relies on the conversion of MTT reagent (#M2128, Sigma-Aldrich) into colorful product formazan by live cells, followed by the measurements of the optical density (OD) of the formazan solution on dimethyl sulfoxide that is proportional to the number of the metabolically active cells. For the calibration of the OD of formazan solution vs. the number of cells, a series of monolayer cell cultures of PANC-1 cells in 96-well plates with gradually decreasing seeded cell numbers (in 24 h after seeding) was added with the same amount of MTT reagent, and, after 4 h incubation at 37 °C, the OD (light absorbance) was measured in a 570 nm spectral band by a multimode plate reader i3X Spectramax (Molecular Devices, LLC., San Jose, CA, USA), with empty wells used as blank controls. The results were corrected for the blank controls by SoftMax Pro Data Analysis software (Molecular Devices, LLC.).

The 3D TECs were analyzed by the MTT assay starting from day 1 since seeding of PANC-1 cells on ALSs and performed on days 1, 3, 7, 14, 21 and 28 of in vitro culture. Before each assay, the media from the wells containing the TECs was removed, the TECs were gently washed twice with PBS to eliminate unattached cells. Next, 3D TECs were aseptically transferred to new 24-well culture plates (3 TECs per experimental time point) to get rid of the cells adhered to the plastic and not to the scaffolds. After double washing with PBS, 500 µL of MTT reagent (0.5 mg/mL in the phenol red free cell culture medium; DMEM/F12; #D6434, Sigma-Aldrich), was added to each well. Then the samples were incubated at 37 °C in a tissue culture incubator for 4 h to allow precipitation of insoluble formazan crystals. After that, the supernatant was carefully collected and 500 μL of DMSO was added to the wells and left for 10–15 min in the dark on a rocking platform at room temperature to dissolve purple formazan crystals. Next, four portions of 100 µL of the dissolved MTT product was taken from each well, transferred to separate wells of a clear 96-well culture plate (#3585, Costar, Corning, Corning, NY, USA) and used for absorbance measurements as described above.

*Live/dead imaging assay.* For the cytotoxicity test, 3D TECs were treated with PLGA–VP NPs, PLGA–VP–PFOB NCs, X-rays and by X-PDT. The viability was measured using a LIVE/DEAD Viability/Cytotoxicity Kit (Invitrogen, #L3224) according to the manufacturer’s protocol. The green (live cells stained with calcein-AM) and red (dead cells stained with ethidium homodimer-1) fluorescence signals were detected using confocal microscopy and calculated by applying the ImageJ software.

### Appendix A.7. Characterization of 3D Model of Metastatic PDAC

Preparation and characterization of ALS and TECs is shown in Figure A7.

The decellularization (DCL) of the chicken liver tissue was accompanied by the expected loss of the native tissue color and gradual achievement of semi-transparency of the organ (Figure A7a–e). At the light microscopy level, DCL led to the removal of cells with preservation of the ECM structure where collagen constituted the most notable component and formed loose meshwork in the parenchymal compartment and denser stromal elements such as blood vessels walls (Figure A7f–g). On day 1 after recellularization, cells individually attached to the ALSs’ surfaces and later formed solid tumor structures (Figure A7h–i). SEM imaging revealed micro- and nanoscale ECM textures of different size on the surface of ALSs, with fine collagen fibres meshes and thick bundles of fibres that were the first attachment sites for the PANC-1 cells (Figure A7j–k). The PANC-1 cells growth dynamics was analyzed by MTT assay (Figure A7l–m). The cell population of TECs was growing for 14 days after seeding, then it stabilized till day 21 and then moderately declined from day 21 to day 28. The highest number of cells per ALS demonstrated the MTT OD values corresponding to 3.5 × 10^5^ cells grown in the monolayer culture.

### Appendix A.8. PLGA–VP–PFOB Dark Toxicity Evaluation in 3D TECs

Figure A8 shows the quantification of live cell viability results for studying the dark toxicity of PLGA–VP–PFOB with various concentration of VP. The cell viability was tested using the MTS assay, and the % cell viability was calculated for different treatments comparing with the control.

## Appendix B

### Graphical Table of Contents

Graphical table of contents. “Bring Your Oxygen” strategy, such as co-delivery of a photosensitizer verteporfin (VP) with an oxygen-carrying molecule PFOB by biocompatible PLGA nanoparticles, enhances the anti-cancer efficiency of radiodynamic therapy (X-ray triggered PDT) by increased in situ ROS generation even under low doses of radiation and in hypoxic environments. This approach may help to overcome treatment resistance of deep hypoxic tumors like advanced pancreatic cancer.
